# Design and Identification of Inhibitors for the Spike-ACE2 Target of SARS-CoV-2

**DOI:** 10.3390/ijms24108814

**Published:** 2023-05-16

**Authors:** Ruan S. Bastos, Lúcio R. de Lima, Moysés F. A. Neto, Maryam, Numan Yousaf, Jorddy N. Cruz, Joaquín M. Campos, Njogu M. Kimani, Ryan S. Ramos, Cleydson B. R. Santos

**Affiliations:** 1 Graduate Program in Medicinal Chemistry and Molecular Modeling, Federal University of Pará, Belem 66075-110, PA, Brazil; 2 Laboratory of Modeling and Computational Chemistry, Department of Biological and Health Sciences, Federal University of Amapá, Macapa 68903-419, AP, Brazil; 3 Laboratory of Molecular Modeling, State University of Feira de Santana, Feira de Santana 44036-900, BA, Brazil; 4 Department of Biosciences, COMSATS University Islamabad, Park Road, Islamabad 45550, Pakistan; 5 Department of Pharmaceutical and Organic Chemistry, Faculty of Pharmacy, Campus of Cartuja, University of Granada, 18071 Granada, Spain; 6 Biosanitary Institute of Granada (ibs.GRANADA), University of Granada, 18071 Granada, Spain; 7 Department of Physical Sciences, University of Embu, Embu 6-60100, Kenya

**Keywords:** COVID-19, new drugs, molecular modeling, antiviral

## Abstract

When an epidemic started in the Chinese city of Wuhan in December 2019, coronavirus was identified as the cause. Infection by the virus occurs through the interaction of viral S protein with the hosts’ angiotensin-converting enzyme 2. By leveraging resources such as the DrugBank database and bioinformatics techniques, ligands with potential activity against the SARS-CoV-2 spike protein were designed and identified in this investigation. The FTMap server and the Molegro software were used to determine the active site of the Spike-ACE2 protein’s crystal structure. Virtual screening was performed using a pharmacophore model obtained from antiparasitic drugs, obtaining 2000 molecules from molport^®^. The ADME/Tox profiles were used to identify the most promising compounds with desirable drug characteristics. The binding affinity investigation was then conducted with selected candidates. A molecular docking study showed five structures with better binding affinity than hydroxychloroquine. Ligand_003 showed a binding affinity of −8.645 kcal·mol^−1^, which was considered an optimal value for the study. The values presented by ligand_033, ligand_013, ligand_044, and ligand_080 meet the profile of novel drugs. To choose compounds with favorable potential for synthesis, synthetic accessibility studies and similarity analyses were carried out. Molecular dynamics and theoretical IC_50_ values (ranging from 0.459 to 2.371 µM) demonstrate that these candidates are promising for further tests. Chemical descriptors showed that the candidates had strong molecule stability. Theoretical analyses here show that these molecules have potential as SARS-CoV-2 antivirals and therefore warrant further investigation.

## 1. Introduction

The coronavirus, a non-segmented virus that acts on single strands of RNA, is a member of the family Coronaviridae and the order Nidovirales. It possesses the type III transmembrane glycoprotein and the envelope protein, which together make up the S proteins in its membrane [1]. Thirty species have previously been identified as being involved in the infection of many different animals, including humans. Many diseases caused by members of this viral family in people are mild, but two beta-type coronaviruses have worsened and spread epidemics. The first of these was SARS-CoV, which was first discovered in 2003 and led to an outbreak that killed 436 people across six countries and was triggered by the acute respiratory syndrome. The outbreak started in Guangdong Province, China, and spread due to travel and tourism in the area. The second was caused by MERS-CoV, the Middle East respiratory syndrome [2], which started in Saudi Arabia in 2012, and by the year 2019, 2468 cases were confirmed with a mortality rate of 35%. Both of these viruses were recognized as global pathogens and became a priority in study, combat, and prevention [3].

When an epidemic started in the Chinese city of Wuhan in December 2019, a new virus belonging to the same family was identified. Initially known as 2019-nCov, later named as a severe acute respiratory syndrome (SARS-CoV-2), it is compared to SARS and MERS but with a high transmission rate [4].

According to research, the angiotensin-converting enzyme 2 (ACE2), which is found in the lung region and primarily controls blood pressure and vasoconstriction, interacts with the virus S protein to cause infection [5]. People with cardiovascular issues are more vulnerable since the virus also affects the heart. COVID-19, therefore, can cause myocardial damage, myocarditis, arrhythmias, and venous thromboembolism, which is why various health complications emerge during therapy for infected patients [6].

Many hypotheses emerged at the onset of the pandemic as researchers strived to find a promising antiviral against SARS-CoV-2. However, due to the low availability of biotechnological resources and limited access to laboratories due to the pandemic, computers have been the main tools for searching new bioactive molecules. Case in point, Smith M. and Smith J. (2020) performed simulations with more than 8000 drugs, using a supercomputer, to evaluate their inhibitory activity against S protein [7].

Virtual screening techniques have been investigated to identify prospective candidates for the treatment of various ailments since they start with already-known molecules, saving money and time. It is crucial to employ several databases that serve as a library of chemicals for this procedure, which are searched using particular criteria such as overlapping chemical structures and electrostatic similarities [8,9].

The molecular docking method is another technique that has been widely used in the development of new drugs. It mainly involves the simulation of bioinformatics between potential drug candidates (the ligands) with the biological receptors of the pathogens (enzymes or proteins), seeking a conformation with better interaction [10,11,12,13].

**Figure 1:**
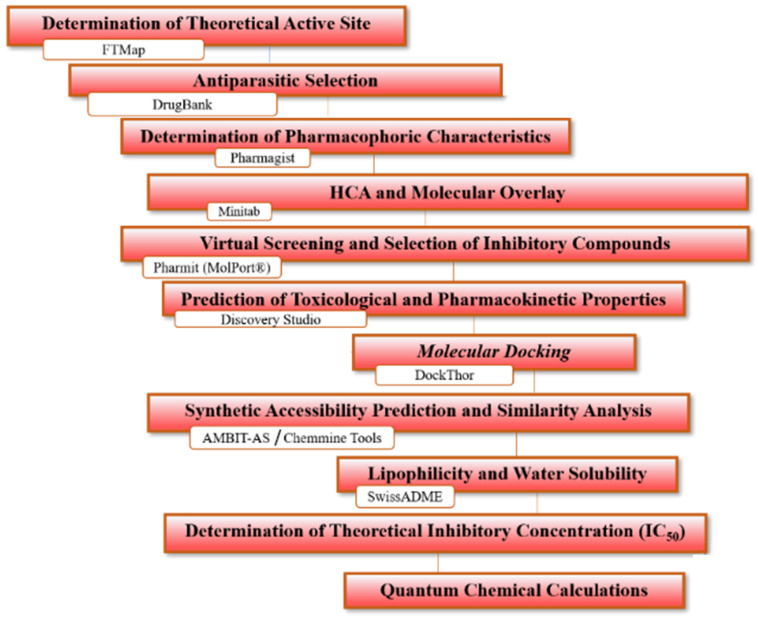
General scheme summarizing the methodological steps.

In this study, the design and identification of ligands with potential activity against SARS-CoV-2 spike protein were performed using virtual screening based on pharmacophores of different structures of antiparasitic drugs to obtain molecules that present binding affinity and pharmacological and toxicological parameters within the expected range for new drugs. The general scheme summarizing the methodological steps in this paper is shown in Figure 1 (refer to Section 3).

## 2. Results and Discussion

### 2.1. Determination of Theoretical Activity Site

For this study, the protein crystal structure of the SARS-CoV-2 spike receptor binding domain bound with ACE2 (Spike-ACE2) (PDB ID: 6M0J) was selected (a design that represents the binding of the spike protein with the host biological receptor). Finding novel methods to identify the activity region was important because the target’s active site has not yet been identified and lacks the complexed ligands required for methods such as redocking.

**Figure 2:**
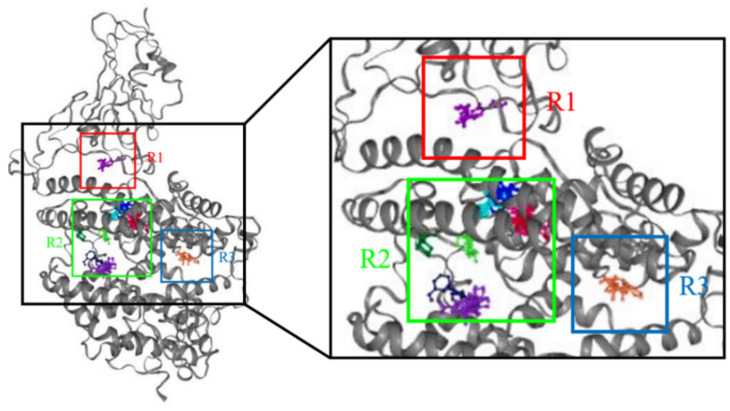
Hotspot regions found by FTMap server for likely active region. The regions R1, R2, and R3 are the possible sites of interaction of the compounds.

At first, the FTMap server pointed out three probable regions of hot spots that can be investigated as the potential active site of the Spike-ACE2 protein (Figure 2). Region 2 presented a more significant number of probe molecules; however, for further confirmation, hydroxychloroquine was docked in all regions and the one in which the binding affinity was the best selected.

Hot spots R2 and R3 were predominant in the region of the ACE2 protein, whereas R1 is concentrated between the protein bindings of the spike protein with ACE2. It was hypothesized that the active region is more likely to be clustered between the two proteins, preventing selectivity for ACE2 alone. Molecular docking supported this hypothesis: Hydroxychloroquine was shown to bind to R1 more readily than in other regions. Therefore, the coordinates of the active theoretical site for this target were determined to be X = −32.770, Y = 24.940, and Z = 6.690.

**Figure 3:**
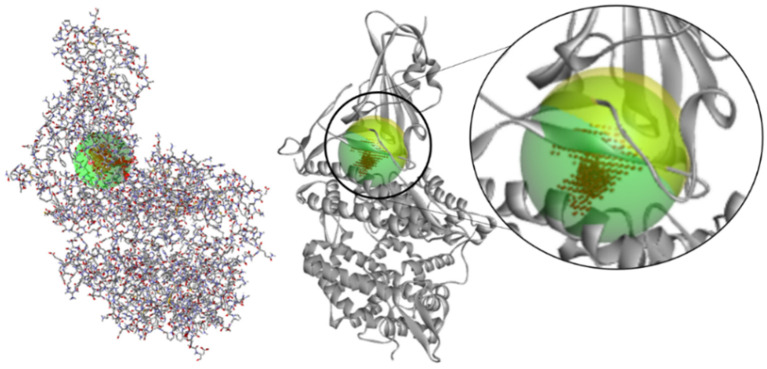
Cavity regions present on the target protein (green detected via Molegro Virtual Docker 5.5 (Odder, Denmark) and yellow region obtained by FTMap WebServer (https://ftmap.bu.edu, Boston, MA, USA)).

To reinforce the hypothesis of the active theoretical site, the Molegro software was used to analyze the cavities present in the protein, and the presence of a cavity in the same R1 region found in the FTMap was noticed, with a difference of ≈1 Å in its coordinates (Figure 3) (X = −33.200, Y = 25.457, Z = 5.543). The active site of enzymes and proteins is typically located in the most excellent cavity pocket present, and this relationship between cavity pockets and their presence has already been examined.

### 2.2. Selection and Optimization of Structures

Based on research demonstrating the inhibitory effects of hydroxychloroquine and chloroquine on SARS-CoV-2 targets, we postulated that compounds with similar structural features and chemical properties would also be potential inhibitors for this target. In this manner, antiparasitic drugs that have already received approval from the relevant agencies were sought to act as the basis for this study.

**Figure 4:**
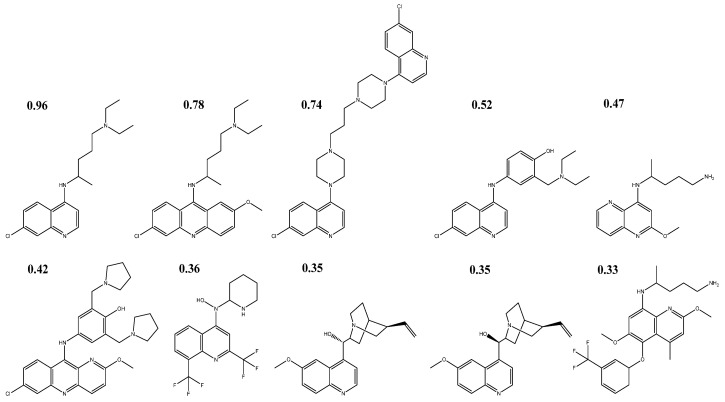
The 2D structures of antiparasitic drugs selected through Tanimoto similarity analysis and their Tanimoto indices.

One hundred antiparasitic drugs were chosen from the DrugBank server and put through a Tanimoto similarity search, which compares the properties of these antiparasitic medications to those of hydroxychloroquine and chloroquine. This is one of the best methods for identifying how similar certain types of molecules are to one another. Only nine of these had an index above 0.32 (Figure 4), which was sufficient for moving on to creating pharmacophoric models and carrying out the virtual screening.

Chloroquine had a Tanimoto index of 0.96 compared to hydroxychloroquine’s structure, while quinacrine, piperaquine, amodiaquine, primaquine, pyronaridine, mefloquine, quinidine, and quinine had Tanimoto indices of 0.78, 0.74, 0.52, 0.47, 0.42, 0.36, 0.35, and 0.35, respectively. Tafenoquine had the lowest Tanimoto indices of 0.33.

Following that, these antiparasitics were put through structure optimization along with hydroxychloroquine and chloroquine to achieve their optimal conformations and prevent errors in the in silico models. Based on the technique of Ferreira et al. (2019) [14], molecular mechanics using MM+ force field through the HyperChem program was used.

### 2.3. Determination of Pharmacophoric Regions

Following the Tanimoto analysis, the molecules were aligned using the Discovery Studio program and transmitted to the PharmaGist web server, with the structure of hydroxychloroquine being used as a pivot. There were three pharmacophoric features produced by alignment. The coordinates of each pharmacophoric property, two aromatic (AR) and one hydrogen bond acceptor (HBC), are shown in Table 1.

The search resulted in a score of 28.062 from the PharmaGist server, the molecules were aligned, taking into account their Tanimoto values, where the reference has a value of 1. As a result, a matrix of pharmacophoric characteristics, atoms (ATM), characteristics (F), spatial characteristics (SF), aromatics (ARO), hydrophobic (HYD), donors (DONN), acceptors (ACC), resulting from the alignment of molecules were generated as shown in Table 2.

### 2.4. Hierarchical Cluster Analysis (HCA) and Molecular Overlay

A structure–activity relationship analysis was carried out utilizing Pearson’s correlation using the physicochemical properties received from PharmaGist based on the Tanimoto index values. Among the observed characteristics, the hydrogen acceptors (ACC) presented the best correlation with the Tanimoto index with a value of −0.564, thus being an important parameter of molecular similarity, followed by the characteristics (F) that present a value of −0.547. Spatial characteristics (SF) showed −0.406, hydrophobic and donors had low values of −0.295 and −0.288, respectively (Table 3).

**Figure 5:**
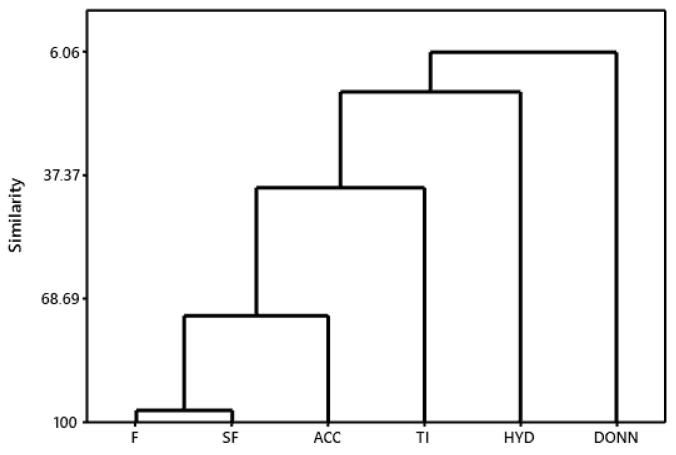
HCA of pharmacophoric characteristics.

Hierarchical cluster analysis (HCA) was performed with the help of Minitab 15 software ( State College, PA, USA). A dendrogram of the pharmacophoric characteristics was generated (Figure 5), which confirmed the Pearson correlation values, demonstrating a greater approximation of ACC with the Tanimoto index (TI) and a departure from the HYD and DONN characteristics.

**Figure 6:**
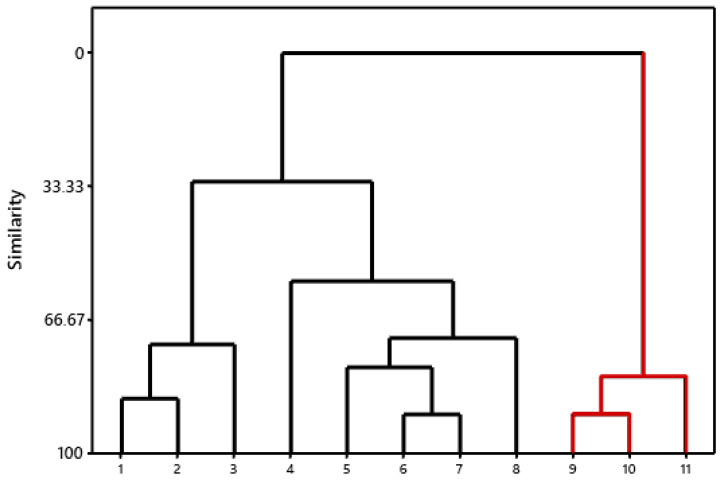
Hierarchical Cluster Analysis Dendrogram (HCA).

As may be observed in two different clusters, the molecules were sorted into groups based on their commonalities (Figure 6). Molecule 1 is hydroxychloroquine, which presents studies of its activity against the target of SARS-CoV-2. Therefore, the molecules of the cluster in which it is found have the potential to exhibit the same response. Compared to hydroxychloroquine, quinidine, quinine, and tafenoquine were in the second cluster and had the lowest likelihood of acting on the target under study.

According to studies by Da Silva Costa et al. (2018) [15] and Cruz et al. (2018) [16], steric and electrostatic forces influence the structural conformation of molecules, especially those with biological functions, so molecular overlap (overlay) was also carried out for the steric (ste) and electrostatic (ele) fields with the aid of Discovery Studio software (Table 4) using 100% as a parameter.

**Figure 7:**
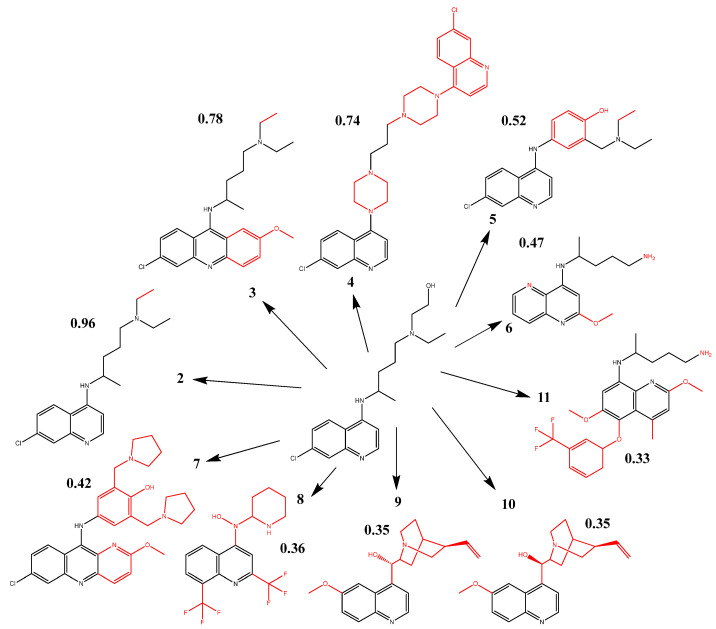
Structural comparison of the reference molecule with the selected ones.

As expected from this investigation, chloroquine provided the best 100ste and 100ele results. For the parameters of 100ste, the three best results of antiparasitics were for quinacrine, quinine, and quinidine, obtaining values of above 80%. Piperaquine, tafenoquine, and amodiaquine performed better for 100ele, with values exceeding 50%. Figure 7 shows the structural comparison of the reference molecule with the other molecules selected for the study.

As a result, molecules 9 and 10 differ somewhat in their hydroxyl stereochemistry. In contrast, molecule 11 contains bonds not found in the reference and trifluoride attached to an aromatic ring. These three structures were the ones that significantly differed from the reference due to their chemical characteristics. The main feature common in all structures is the presence of quinoline (C_9_H_7_N), a scaffold consistent with the pharmacophores obtained. In addition to having a bonded chlorine atom, molecules 3, 4, and 5 have larger Tanimoto indices and improved structural closeness, as seen in the HCA cluster. Molecule 3 is the closest to the chemical structure of hydroxychloroquine (except for chloroquine).

### 2.5. Virtual Screening

The virtual screening took place on the Pharmit online server using the coordinates obtained from the pharmacophore. Molecular weight (MW), rotatable bonds (RB), partition coefficient (LogS), polar surface area (PSA), number of aromatic rings (AR), hydrogen bond acceptors (HBA), and hydrogen bond donors (HBD) are some of the defined reduction filters that were used to limit the resulting structures to this range of defined values. These filters were selected based on the highest and lowest value presented by all drugs (see Table 5).

**Figure 8:**
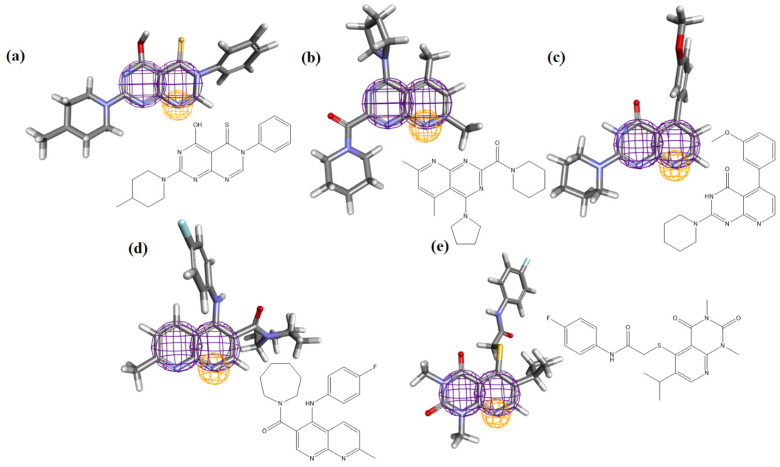
Molecules resulting from the study and pharmacophoric region: (**a**) Ligand_003, (**b**) ligand_013, (**c**) ligand_033, (**d**) ligand_044, (**e**) ligand_080.

The MolPort database was used, and it produced a total of 2000 molecules while accounting for the lowest values of RMSD. All structures showed an RMSD of 0.17, demonstrating the model’s effectiveness. Figure 8 illustrates the properties of the pharmacophores for the five compounds obtained from the screening procedure and accepted in this study to illustrate the behavior of the pharmacophore regions.

### 2.6. Prediction of Toxicological and Pharmacokinetic Properties

**Figure 9:**
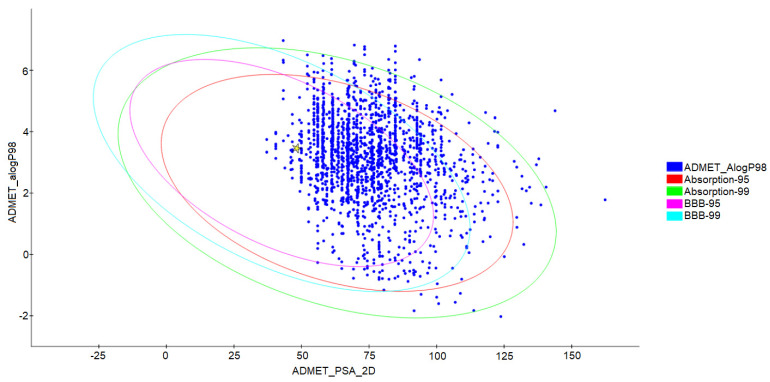
ADMET graph relating the polar surface area to the calculated ALogP98 values, the ellipses represent the 95% and 99% confidence limits, and the highlighted star means the drug hydroxychloroquine.

The 2000 molecules resulting from the screening process were submitted to evaluate their absorption properties, distribution, metabolism, excretion, and toxicity (ADMET). A graph (Figure 9) was obtained based on the 95% and 99% confidence values for the blood–brain barrier (BBB), human intestinal absorption (HIA), polar surface area (PSA), and lipid solubility (LogP), which resulted in 1397 molecules for the subsequent phases.

Hydroxychloroquine, represented as a star in the graph, was used as a control molecule for pharmacological characteristics. The ellipses show the 95% and 99% confidence-bound space for promising candidates. Those found in this region have features similar to compounds with a capacity of ≥90% to be absorbed, a value of <140 for PSA, and <5 for ALogP98. Only candidates within the 99% confidence limit are selected [17].

The results of the ADMET predictions can be seen in Table 6. The 11 compounds selected together with hydroxychloroquine were evaluated. Ligand_020, ligand_035, ligand_063, and ligand_080, as well as the control drug, showed false negative results for binding to plasma proteins. The others showed false positives and may be highly linked to PPB. This is related to lipophilicity, whereby the higher the lipophilicity, the stronger the link with PPB. In this way, inferences on how these molecules can circulate in the bloodstream can be made. In the same way, PPB properties are related to toxicity that can cause severe unwanted consequences [18].

All ligands showed false positive values for hepatotoxicity as with the control drug. This is one of the main drawbacks of synthetic medications, indicating that they can impair liver function or induce liver disorders when used in large doses over an extended period. However, biological studies are needed to prove such data. None of the candidates showed the potential to bind CYP2D6, which is present in 2% of the hepatic CYP content and is responsible for the metabolism of several drugs. Its binding can affect drug metabolism and impair the desired effect [19].

All ligands showed poor or very poor solubility, with emphasis on hydroxychloroquine and ligand_020. Regarding blood–brain barrier penetration, ligand_020 had low penetration, the control drug and ligand_003, ligand_005, and ligand_044 exhibited strong penetration, and the others were just average.

Parameters a, b, and c in Table 6 are classified based on a compound with a high binding index (≥90%) with a Bayesian score with a value of −2.209, classified as false positive (true) or false negative (false). The BBB controls the flow of solutes from the blood to the brain and facilitates molecular communication between the central nervous system and other tissues. A high penetration for these candidates is ideal as studies indicate that the SARS-CoV-2 virus can affect brain cells [20].

All molecules in this study showed good intestinal absorption, parameters responsible for drug transport and release. The candidates also underwent in silico toxicity testing using FDA models of male and female rats and mice (see Table 7).

None of the candidates showed carcinogenic potential. Hydroxychloroquine had mutagenic potential in the Ames tests, but the study candidates did not, indicating that the chosen compounds may have fewer side effects.

Regarding skin irritation, hydroxychloroquine, ligand_005, ligand_013, ligand_044, and ligand_080 showed no irritation, ligand_003, ligand_020, ligand_033, ligand_035, ligand_049, and ligand_063 showed mild irritation. Regarding eye irritation, hydroxychloroquine, ligand_005, and ligand_035 showed severe values, ligand_003, ligand_013, ligand_020, ligand_044, ligand_063, ligand_080, and ligand_086 had moderate values, while ligand_033 and ligand_049 were mild. Only ligand_020 presented a degradable parameter for aerobic biodegradability. After these toxicological analyses, 99 candidates were approved for molecular docking studies.

### 2.7. Molecular Docking

Twelve compounds were chosen for the molecular docking investigation after the ADMET evaluation, including hydroxychloroquine, which served as a reference. The simulation was run on the Dockthor server, utilizing coordinates found while determining the hypothetical active site (X = −32.770, Y = 24.940, and Z = 6.690). The molecular target in this study was the structure of the SARS-CoV-2 spike receptor bound with ACE2 (PDB ID: 6M0J). To observe binding affinity values and molecular interactions, see Table 8 vide infra.

**Figure 10:**
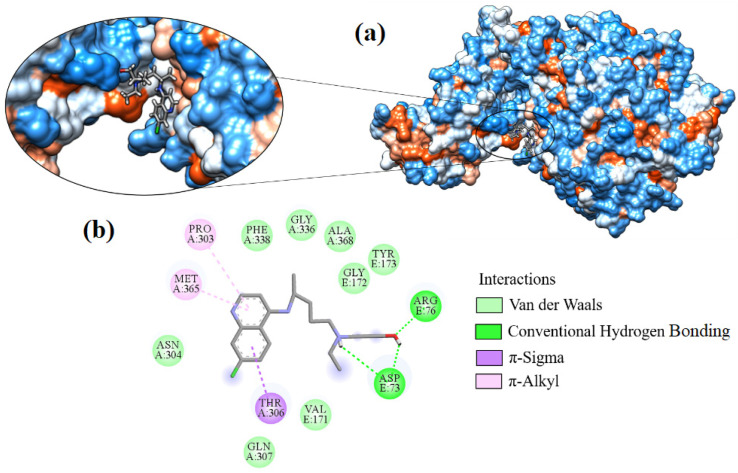
Interaction of hydroxychloroquine with Spike-ACE2, (**a**) 3D contact surface, (**b**) 2D interactions diagram.

Hydroxychloroquine showed a binding affinity of −7.595 kcal·mol^−1^ to the target and reacted with two residues through hydrogen bonds. The hydrogens of both amines (NH_2_) of the ARG76 residue interact with the ligand’s hydroxyl (-OH). The oxygen atom of ASP73 interacted with the hydroxyl and the amine group of hydroxychloroquine (Figure 10).

The stability of the ligand within the protein and its binding affinity are significantly influenced by hydrogen bonds, which are of enormous relevance. π-sigma interactions also occur between the aromatic ring with residue THR306 and π-alkyl with the pyridine fragment (C_5_H_5_N) with residues PRO303 and MET365. Amino acids ALA368, ASN304, GLN307, GLY172, GLY336, PHE338, TYR173, and VAL171 interact with the ligand hydrophobically.

**Figure 11:**
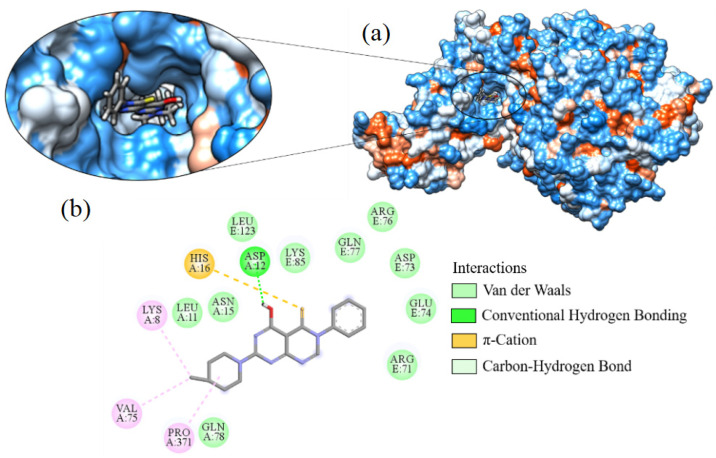
Interaction of ligand_003 with Spike-ACE2, (**a**) 3D contact surface, (**b**) 2D interaction diagram.

The best binding affinity value was observed for ligand_003 with a value of −8.645 kcal·mol^−1^. There was only one hydroxyl hydrogen bond for this ligand with residue ASP12 (Figure 11). Other interactions occurred whereby the residue HIS16 formed a π-sulfur interaction with the sulfur present in the structure. The piperidine fragment (C_5_H_11_N) forms a π-alkyl-type interaction with the residues LYS8, PRO371, and VAL75. Additionally, hydrophobic interactions with the residues ARG71, ARG76, ASN15, ASP73, GLN77, GLN78, GLU74, LEU11, LEU123, and LYS85 were observed.

**Figure 12:**
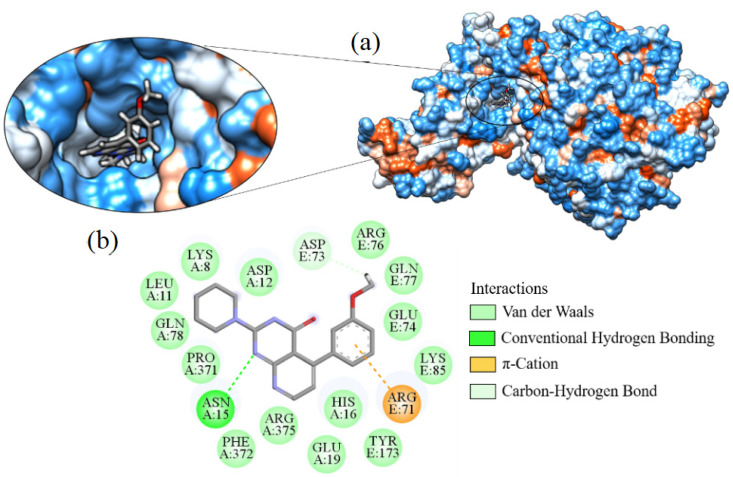
Interaction of ligand_033 with Spike-ACE2, (**a**) 3D contact surface, (**b**) 2D interaction diagram.

Ligand_033 indicated a binding affinity of −8.303 kcal·mol^−1^ when bound to the SARS-CoV-2 target structure. This interaction resulted in a hydrogen bond between the amine hydrogen of the ASN15 residue with the pyrimidinone nitrogen (C_4_H_4_N_2_O) present in the ligand. Interaction of the ligand with ARG71 was through a π-cation-type bond (Figure 12). This was the monopole type (cation), where a large electrostatic force occurs due to the abundance of electrons in the system, being a common interaction in nature. The residue ASP73 formed a carbon–hydrogen interaction with the methyl hydrogens on the ligand. The other interactions were hydrophobic with residues ARG375, ARG76, ASP12, ASP73, GLN77, GLN78, GLU19, GLU74, HIS16, LEU11, LYS8, LYS85, PHE372, PRO371, and TYR173.

**Figure 13:**
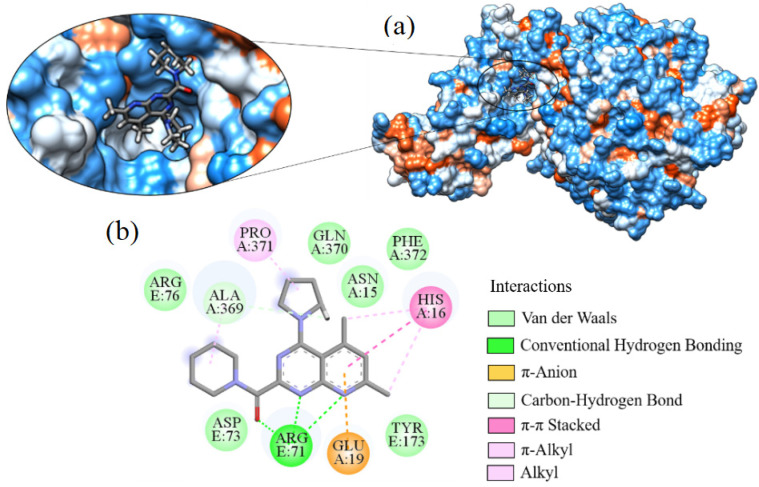
Interaction of ligand_013 with Spike-ACE2, (**a**) 3D contact surface, (**b**) 2D interaction diagram.

For ligand_013, hydrophobic interactions occurred with residues ALA369, ARG76, ASN15, ASP73, GLN370, GLU19, HIS16, PHE372, PRO371, and TYR173. It also formed a hydrogen bond between one of the amine hydrogens of the ARG71 residue with the pyridine nitrogen of the ligand (see Figure 13). A carbon–hydrogen bond occurred between residue ALA369 and the pyrrolidine fragment (C_4_H_9_N) and an π-alkyl type of bond with pyridine. HIS16 and PRO371 residues also made π-alkyl bonds. These interactions occur between the electron cloud and an alkyl group. HIS16 also formed a stacked π–π interaction. These stacked π–π interactions are of the dipole–dipole type occurring due to the electrostatic attraction of the electrons of the aromatic ring. They play an important role in the stability of proteins. GLU19 makes a π-anion-type bond, occurring mainly in electron-deficient aromatic rings. This interaction is rare in biological systems as amino acids tend to repel nearby negative charges.

**Figure 14:**
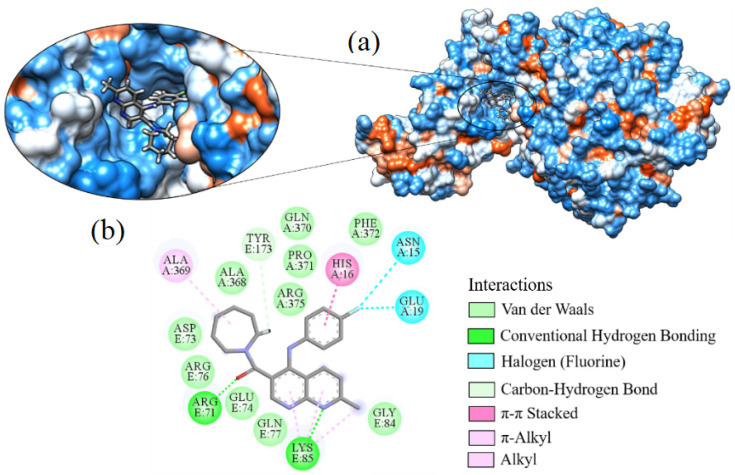
Interaction of ligand_044 with Spike-ACE2, (**a**) 3D contact surface, (**b**) 2D interaction diagram.

Ligand_044 showed a binding affinity of −7.749 kcal·mol^−1^ and formed two hydrogen bonds between the oxygen of the ligand and hydrogen of the amine present in residue ARG71 and residue LYS85 (Figure 14). Hydrophobic interactions occurred with 16 amino acid residues, ALA368, ALA369, ARG375, ARG76, ASN15, ASP73, GLN370, GLN77, GLU19, GLU74, GLY84, HIS16, LYS85, PHE372, PRO371, and TYR173. The ALA369 and LYS85 residues also form π-alkyl interactions with the azepane fragment (C_6_H_13_N) and the pyrimidines. The HIS16 residue forms a π–π-type interaction stacked with the aromatic ring, while the amino acid TYR173 forms a bond of the carbon–hydrogen type. There are also halogenated interactions of fluorine with the amino acids ASN15 and GLU19. The homeostasis of various physiological processes is directly influenced by fluorine. Halogens in the ligands may improve drug binding affinity and selectivity by acting as an electron acceptor and directly participating in the interaction with biological receptors.

**Figure 15:**
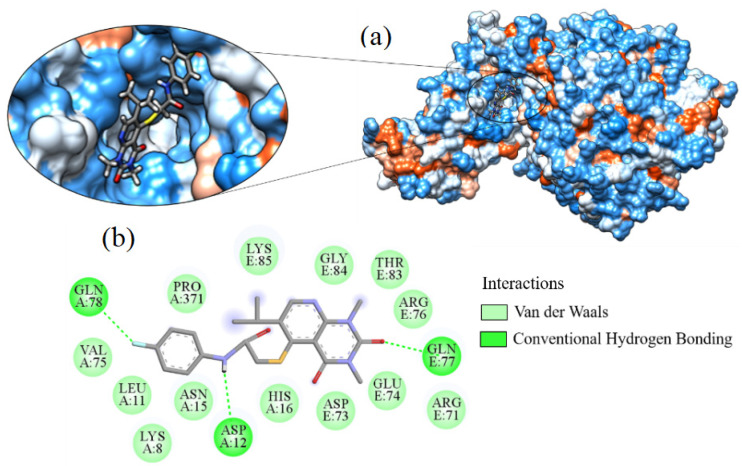
Interaction of ligand_080 with Spike-ACE2, (**a**) 3D contact surface, (**b**) 2D interaction diagram.

The binding affinity of ligand_080 was −7.690 kcal·mol^−1^. The ligand generated 14 hydrophobic interactions with the Spike-ACE2 target with residues ARG71, ARG76, ASN15, ASP73, GLN78, GLU74, GLY84, HIS16, LEU11, LYS8, LYS85, PRO371, THR83, and VAL75. Three hydrogen bonds were formed between the oxygen atom of ASP12 and the nitrogen in the ligand structure, the amine nitrogen of GLN77 and ligand oxygen, and one with residue GLN78 (Figure 15).

**Figure 16:**
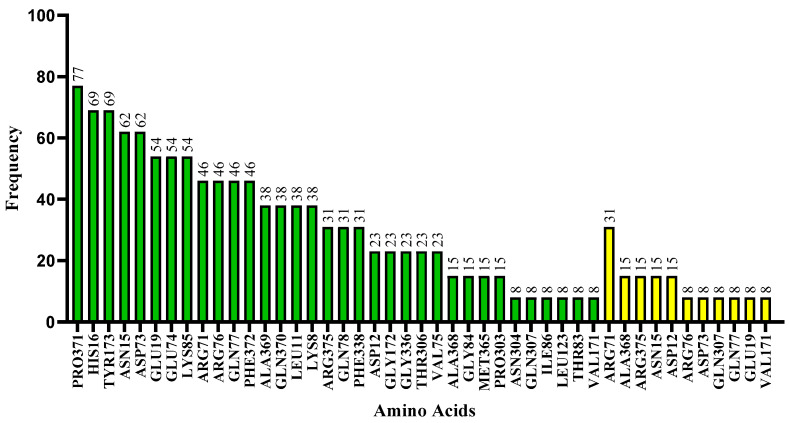
Frequency of amino acids present in ligand in contact with the SARS-CoV-2 target. Color system: Hydrophobic interactions (green) and hydrogen bonds (yellow). The numbers above the bars indicate the percentage of contacts for each amino acid residue.

By examining the docking results for all 12 ligands and hydroxychloroquine, it was feasible to confirm that more hydrophobic interactions (34 residues) than hydrogen bonds (11 residues) were used to make contact between the ligands and the Spike-ACE2 protein (see Figure 16).

**Figure 17:**
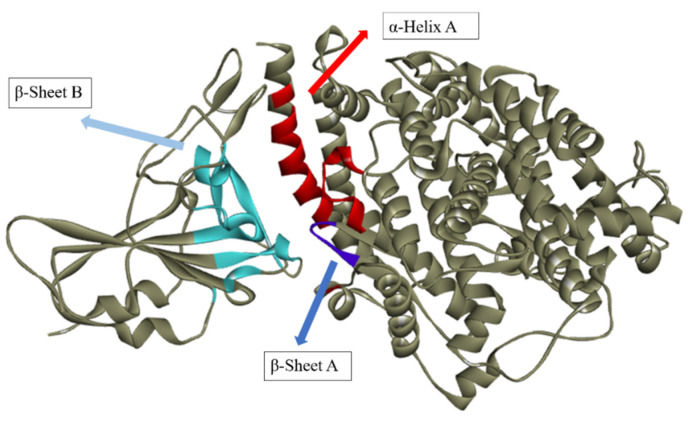
Predominant regions of amino acid residues that interacted between the ligands and the Spike-ACE2 protein.

Interacting residues are predominant between the region of α-helix A and β-sheet A of ACE2, and the region of β-sheet B of the spike protein (Figure 17). The most frequent residue was PRO371, which was present in β-sheet A in 77% of the cases. Next in frequency were residues HIS16 from the α-helix and TYR173 found in β-sheet A, both of which were detected in 69% of the instances.

When all interactions of each ligand are taken into account, the residue with the highest frequency for hydrogen bonds was ARG71 with 31% in β-sheet B, which is favorable to what is anticipated because this region comes from the spike bond, followed by residues ALA368 (β-sheet A), ARG375 (β-sheet A), ASN15 (α-helix A), and ASP12 (α-helix A) with 15%. All amino acid residues that established hydrogen bonds also interacted through hydrophobic interactions.

Arginine, a positively charged guanidinium group with pH 7.0, is a residue responsible for catalyzing several enzymes. Its main action is found in the synthesis of nitric oxide. The interaction of the ligand with this residue may favor the inhibition of the spike protein, as it is responsible for decreasing the bioavailability of nitric oxide, and responsible for vasodilation and blood pressure control.

### 2.8. Synthetic Accessibility Prediction and Similarity Analysis

Following the ADMET examination, the 99 compounds were put through the synthetic accessibility (SA) test, which filtered out the molecules that were not likely to be synthesized. The AMBIT-AS program was used to predict SA, and its algorithm computes scores ranging from 0 to 100 based on several criteria relating to the chemical properties of the structures, where a molecule with great ease of synthesis has a value of 100 [21]. Hydroxychloroquine was used as a standard and had an SA value of 69.792. Here, we only selected the molecules that achieved values greater than this reference molecule, indicating that they were highly likely to be synthesized (see Table 9).

**Figure 18:**
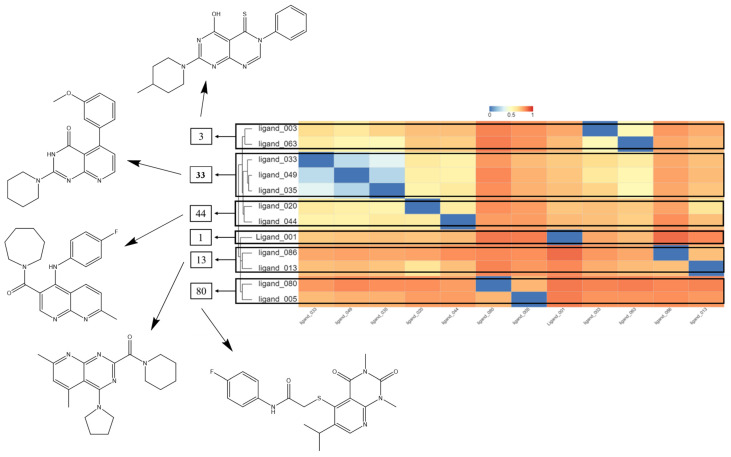
Heatmap of the hierarchical cluster showing the molecular similarity separated into different clusters and the most promising structures for further studies.

After making accessibility predictions, the Chemmine Tools server was used to conduct similarity research. The structures were divided into clusters according to their individual physical–chemical and structural features. They were also divided into clusters, and five molecular structures—one for each cluster—and hydroxychloroquine was chosen to proceed with this study. The selection was based on the best binding affinity values, taking into account that there is a chance that they will have similar biological activity in in vitro experiments if they are in the same cluster (see Figure 18). Ultimately, 12 interesting compounds were chosen for further study, and molecular docking studies were run on each.

### 2.9. Lipophilicity and Water Solubility

The SwissADME server was employed to ascertain the selected molecules’ lipophilicity and water solubility characteristics. These properties have an impact on the kinetics of how drug candidates act, making them crucial in the process of identifying novel medications. The octanol–water partition coefficient (LogPo/w), which results from the concentration of a molecule in its neutral form in the organic and aqueous phase, is used as a benchmark for lipophilicity [18]. Lipophilicity affects the solubility and permeability properties of membranes in a way that can change the molecules’ ADMET profiles and directly reflect the molecular forces active between the two phases. For novel compounds, LogP < 5 is a desirable number [18].

The SwissADME server allows the investigation of five distinct values, XLOGP3 which uses a database of molecules as a basis for identifying LogP values, using 87 different types of atoms and two groups of correction factors, WLOGP that uses a fragmented method proposed by Wildman and Crippe through atomic contributions, MLOGP that uses 13 descriptors of hydrophobic, hydrophilic, unsaturated bond, and other atoms based on the linear relationship to determine their values, SILICOS-IT which uses seven descriptors in its approach in a hybrid system based on the FILTER-IT software source code, and iLOGP that uses the energies of free water and n-octanol solvations using a generalized Born equation and solvent accessible surface area (GB/AS) approach.

**Figure 19:**
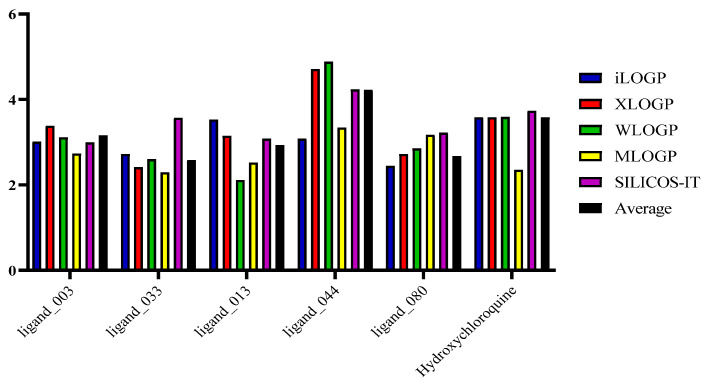
Predicted lipophilicity values (LogPo/w) of promising candidates and hydroxychloroquine (control).

The five selected structures and hydroxychloroquine were passed through the SwissADME server to obtain their lipophilicity values and an average of the results (Table 10 and Figure 19). As suggested by Lipinski for compounds with optimal lipophilicity, all molecules displayed values below 5, making them desirable. Ligand_044 presented a higher average than the control drug, which could be associated with the presence of the halogen (F) in its structure. Hydroxychloroquine, the standard, also has a halogen (Cl) registered as the second highest value. The third highest result was ligand_003, possibly due to the double bond with sulfur (S). The values obtained in these studies ranged from 2.58 to 4.22, characterizing them as highly lipophilic, meeting the criteria required for drug candidates.

Water solubility (LogS) is a crucial investigational factor since it relates to the route of delivery of drug candidates, particularly when addressing oral and parenteral routes, due to the concentration required for the candidate’s circulation in the distribution system. The usage of molecules with low solubility has proven problematic since their insoluble classification affects how they behave in the circulatory system. Many non-polar alkene and alkyne bonds in the structures affect the compounds’ solubility since they prevent the compounds from dissolving in water.

The molecules are more soluble in water when a hydroxyl group is present, and a ketone functional group has a similar effect because of the potential for molecular interactions. With the help of SwissADME, three methods—the ESOL method, the Ali method, and the SILICOS-IT technique—were designed for this study to determine water solubility [22].

**Figure 20:**
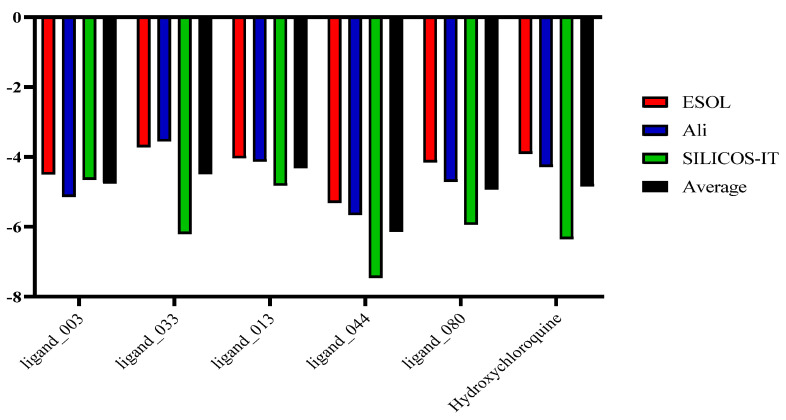
Predicted water solubility values (LogS) of promising candidates and hydroxychloroquine (control).

In terms of solubility, ligand_044 and hydroxychloroquine produced the best results. According to the SILICOS-IT parameter, ligand_044 (−7.46) and hydroxychloroquine (−6.35) have low solubility. A moderate solubility displays a LogS between −4 and −6, while a high solubility displays a LogS from −2 to −4. Except for ligand_013 and ligand_044, all others showed moderate water solubility. However, they are still potential candidates for oral or parenteral administration (Table 11 and Figure 20).

### 2.10. Molecular Dynamics Studies

**Figure 21:**
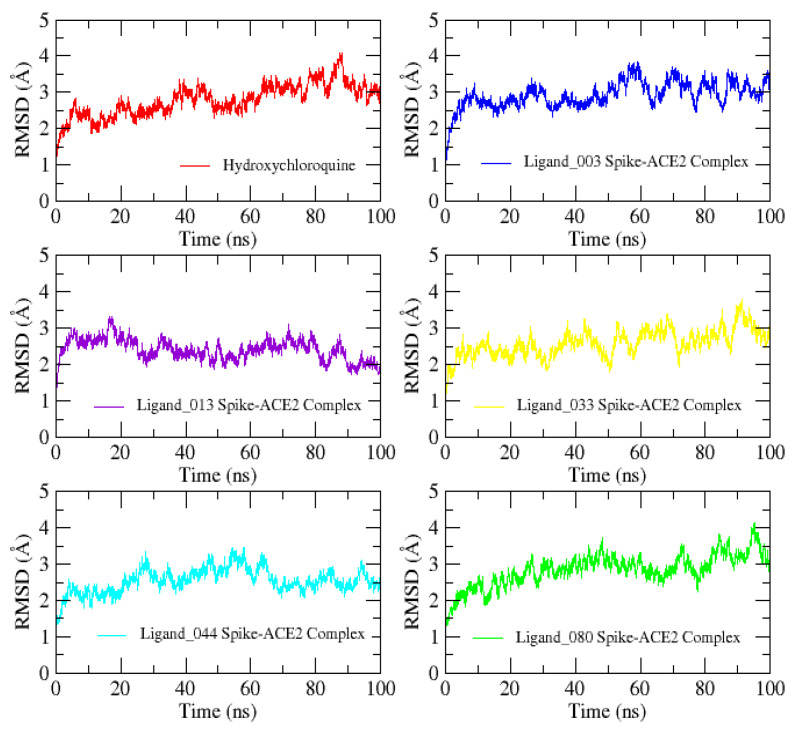
Root mean square deviation (RMSD) plot of control and selected screened compounds in complex based on Cα in complex with spike protein.

To understand the stability and binding affinity of ligands complexed in the spike protein binding site, we performed an MD simulation. Protein conformational changes disclosed its function, and MD is crucial to comprehending biological activity. After MD simulation, trajectory data were analyzed in terms of RMSD, RMSF, radius of gyration, VMD, and R-code. RMSD based on Cα of spike protein with its complexes is plotted in Figure 21.

The RMSD plot shows very stable curves that indicate the system’s stability during the trajectory. The average RMSD of all ligands has similar curves compared with the control compound (hydroxychloroquine) and ranges from 2–4 Å. The effect of conformational change is more (2–4 Å) in the control compound, ligand_003–Spike-ACE2 complex, and ligand_080–Spike-ACE2 complex. Ligand_033–Spike-ACE2 complex was moderate (2–3.5 Å) and ligand_013–Spike-ACE2 complex and ligand_044–Spike-ACE2 complex (2–3 Å) were lowest. Low variation indicates that ligands are still interacting within the binding cavity of the spike protein during the simulation period, despite minor conformational changes.

**Figure 22:**
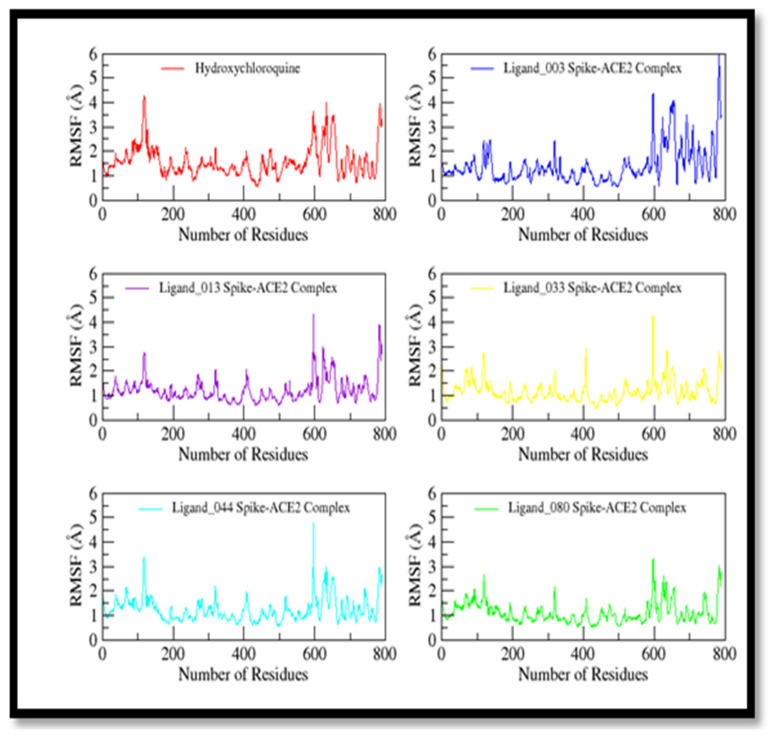
Root mean square fluctuation (RMSF) plot of control and selected screened compounds in complex based on Cα in complex with spike protein.

The Cα-based RMSF was observed, as shown in Figure 22. The overall RMSF curve of all the complexes is stable from the central region, especially the binding site region, while fluctuating at the loop region, including residues from 600 to 800. The loop region shows more flexibility due to the high degree of freedom. The average fluctuation is from 1–2 Å at the central residues, while the loop region shows fluctuation up to 5 Å that shows the system’s overall stability. Ligand_033–Spike-ACE2 complex shows a little fluctuation at residual position 400 during simulation, but that residue does not share a binding cavity region, so its variation does not affect binding strength of the complex.

**Figure 23:**
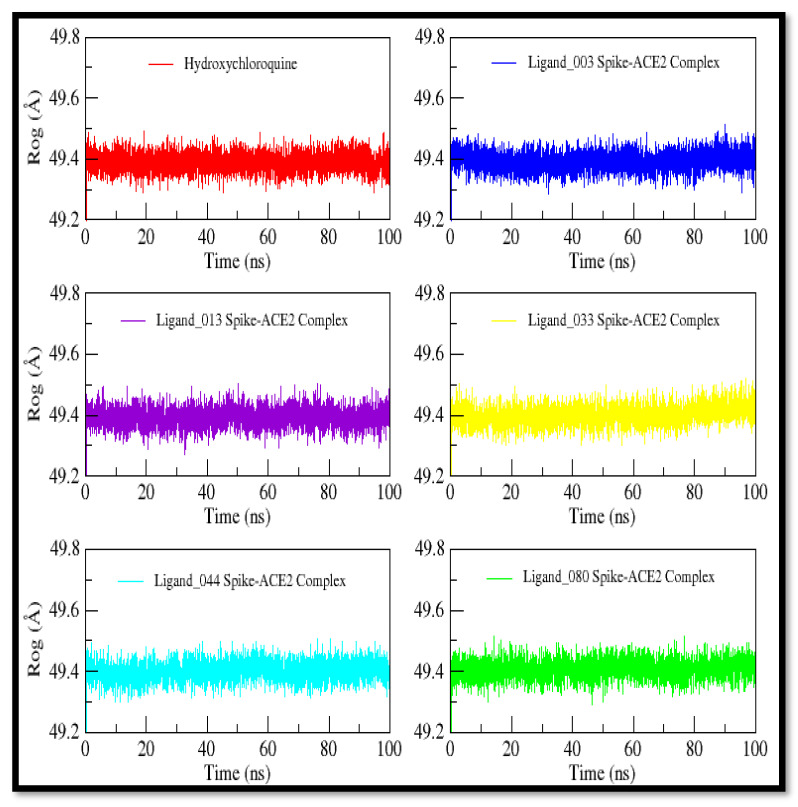
The radius of gyration plot of control and selected screened compounds in complex based on Cα in complex with spike protein.

The compactness of the protein is shown by the radius of gyration, as indicated in Figure 23. Based on mean values that range from 49.3 to 49.6 Å, it can be concluded that the protein is still compact and that no significant modifications are seen when spike protein is in the presence of its ligands. The constant mean value shows that protein is stable and there are no major conformational changes in spike protein. According to RMSD, RMSF, and gyration radius, the ligands could stabilize the spike protein binding site during the MD trajectories.

#### Binding Free Energy Calculation

The binding energy of ligand_003, 013, 033, 044, and 080 in the spike protein binding site was calculated by MMPBSA methods, and obtained results are summarized in Table 12. The ∆G_total_ indicated that all the complexes had excellent simulation-based binding energy values that were near to those of the control compound. The MMPBSA result ranks the compounds ligand_44 < ligand_33 < hydroxychloroquine (control) < ligand_03, ligand_80 < ligand_013.

Moreover, different components of the energies, vdW, electrostatics, polar solvation energy (EPB), and non-polar solvation energy (ENPOLAR), all show good binding strength. In control, compound electrostatics is a major energy contribution, whereas in other compounds, vdW is dominant over other energy terms.

The fact that the polar solvation energy (EPB) remained notably unfavorable also indicates that vdW plays a key role in the binding of ligands to spike protein. Ligand_044 shows a stronger and more significant free energy value than other complexes and has the highest vdW, electrostatic, and EPB values. While all compounds had positive binding interactions and significant binding energy values in the protein binding site, ligand_013 had the lowest ΔG_total_ due to the lowest electrostatic contribution.

### 2.11. Determination of the Theoretical Mean Inhibitory Concentration (IC_50_) 

The mean inhibitory concentration (IC_50_) is a measure used to assess the ability of a compound to inhibit half of the biological activity in a specific target from an initial amount [23,24]. The IC_50_ measurement value has always been one of the limitations of theoretical and in silico approaches. In this work, we used a mathematical equation proposed by Hopkins et al. (2014) [25], in which the initial formula was deduced and rearranged to estimate the pIC_50_ value (Table 13).

As is already known for pIC_50_ values, the higher the value obtained, the more active the compound studied, while the IC_50_ value is the opposite, i.e., the lower its value, the better the activity. With a predicted IC_50_ value of 0.459 µM, ligand_080 has the potential to be the most active. The values discovered were 0.586 µM and 0.663 µM for ligand_013 and ligand_033, respectively. For ligand_003, the highest value was 2.371 µM. The value of hydroxychloroquine was inferior to all the predicted values for the ligands.

### 2.12. Quantum Chemical Calculations

Chemical descriptors play a crucial role in the chemistry and pharmacology of the interaction between the ligand and the macromolecule. The HOMO and LUMO energies and their gap show the charge transfer potential that affects the molecule’s bioactivation and the electrostatic potential connected to the binding of ligands to the surface of the macromolecule [26].

**Figure 24:**
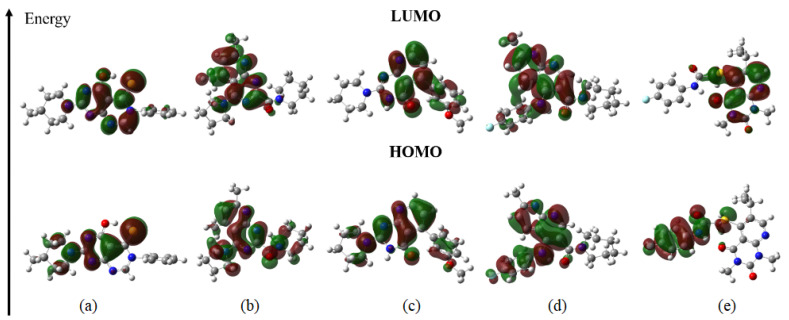
Molecular orbital HOMO and LUMO for the 5 candidates, (**a**) ligand_003, (**b**) ligand_013, (**c**) ligand_033, (**d**) ligand_044, and (**e**) ligand_080.

The five candidate compounds were subjected to chemical molecular optimization calculations using the Gaussian 09 program. As a result, the highest occupied molecular orbitals (HOMOs) and lowest unoccupied molecular orbitals (LUMOs) of each structure, as well as their gap values, were determined for each compound (Figure 24). The HOMOs and LUMOs are of great importance in the study of the reactivity and stability of molecules, where the HOMO energy demonstrates the ability of a molecule to donate its electrons and the LUMO energy to accept electrons.

The HOMOs are predominant mainly over the pyridine and pyrimidine fragments of the structures, except ligand_080 where this fragment did not present a molecular orbital, a functional group known to perform hydrophobic interactions in its aromatic rings, which was observed in molecular docking studies. The presence of these orbitals explains the π-anion bond present in the aromatic ring of ligand_013. For ligands that contain sulfur, it is possible to see the orbital density on this atom favoring the π-sulfur bond as observed in docking studies, as well as the fluorine atoms that formed the halogen bonds with the spike target. The oxygen atoms also presented HOMOs except for the hydroxyl in ligand_003 and the ketone in ligand_080. The eigenvalues were all very similar, showing that their electron-donating potential is similar.

The HOMOs and LUMOs are localized on the pyridine and pyrimidine fragments with the exception of some fragments that mainly have rings linked to fluorine. For the LUMO values, it is possible to see a difference where ligand_033 had the lowest value of −1.638 eV and ligand_080 the highest of −2.699 eV. The orbitals on ligand_033 favored the π-cation bond, which is explained by the density of the orbital on the aromatic ring next to the oxygen of the ether.

These orbitals are of great importance in biological interactions, mainly influencing the binding affinity of ligands with targets through interactions, as seen in molecular docking. The HOMO and LUMO data were also important in the calculation of the descriptors gap energy (∆E), ionization potential (IP), electron affinity (EA), electronegativity (χ), chemical potential (μ), chemical hardness (η), softness (σ), and electrophilicity (ω), see Table 14.

The values of ∆E are related to the chemical reactivity of the compound and are obtained from the difference in the energy of HOMO and LUMO, where a smaller gap indicates the low stability of the structure. According to the data collected, ligand_013 has the best stability among the potential ligands, attaining a value of 4.380 ev. The others presented values that vary between 3.449 eV and 4.434 eV and this demonstrates that all candidates have excellent chemical stability, favoring the stability of the ligands within the spike target substrate.

The IP is related to the ability of the structures studied to donate electrons. Since it relates to the transfer of electrons present in the structure, it is a parameter that may be used to determine the antioxidant potential of the candidates evaluated. All compounds had close values, with the highest value of 6.334 for ligand_013 and the lowest value of 6.071 for ligand_033. This demonstrates that all compounds have an antioxidant power and are great candidates for anti-SARS-CoV-2 action.

EA is related to the ability of the ligands to accept electrons. Its relationship to antioxidant activity is unknown. However, it has a characteristic that is the opposite of IP. Its connection to the potential for reduction and its capacity to neutralize free radicals is apparent. All candidates had a low value of EA, as expected. The lowest value observed was for ligand_033 of 1.638 eV and the highest for ligand_080 (2.699 eV), demonstrating the low potential for accepting electrons and confirming its antioxidant power.

The μ is related to the energetic change of the system through the electronic trend influencing the molecule’s electron density with the target substrate. The highest observed value was −4.423 eV for ligand_080, and the lowest was −3.855 eV for ligand_033. The values were approximate for all ligands showing a nucleophilic (electron donor) and electrophilic (electron acceptor) character indicating their charge transfer capacity.

The η is related to the molecular stability of compounds following the maximum hardness principle (MHP), which indicates that hard molecules will be less reactive than soft molecules due to resistance in charge transfer. All compounds showed similar values indicating that the molecules have low hardness and are reactive. The acceptance of loads and measures of the degree of molecular activity characterize the parameter σ. The studied ligands showed a high smoothness.

The value of ω relates to the direction in which the charge transfer occurs and its electrophilic potential, that is, the molecule’s stability when acquiring an extra electron density. The molecules studied can thus be classified as strong electrophiles.

**Figure 25:**
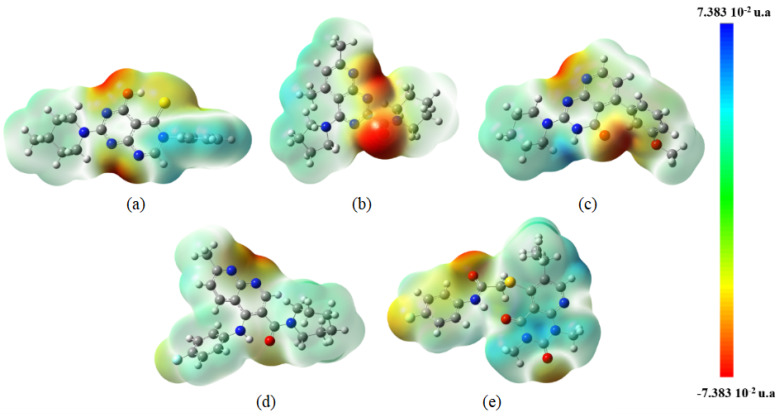
Electrostatic potential map of the 5 candidate structures, (**a**) ligand_003, (**b**) ligand_013, (**c**) ligand_033, (**d**) ligand_044, and (**e**) ligand_080.

The values of χ are related to the attraction of electrons in the molecule. Another piece of information that can be observed is the molecule’s electrostatic potential, which can be observed in Figure 25. The ligands were shown to be electronegative, which provides the exchange of electrons with the substrate, favoring electrostatic attraction and increasing the force of interaction.

The electrophilic and nucleophilic areas of each ligand’s structure can be observed using the molecular electrostatic potential, with the most electronegative region being represented by red and the least electronegative part by blue. These areas are connected to the area where the majority of a compound’s molecular interactions occur. In ligand_003, the most electronegative region is close to oxygen and sulfur, the same region that presented the hydrogen bond with the spike protein. The least electronegative region was at the ends of the molecule in the aromatic ring and pyridine. For ligand_013, there was also a higher electronegative concentration of oxygen; as observed in the molecular docking in this region, hydrogen bonds to the spike protein occurred.

Along with the other ligands, ligand_033 displayed the electronegative region on the structure’s two oxygens and the opposite region on the pyridine and pyrimidine nitrogens, the region in which the hydrogen bonds took place. While ligand_044 displayed an electronegative density in the nitrogen and oxygen regions, both sites for hydrogen bonding, fluorine did not exhibit a high electronegativity for this ligand. For ligand_080, the most electronegative region is found on the oxygens and fluorine, as expected, the regions where hydrogen bonds occurred in molecular docking.

## 3. Materials and Methods

### 3.1. Determination of Theoretical Active Site

With the use of the FTMap server, identifying the potential region of biological activity was investigated (https://ftmap.bu.edu/, accessed on 1 January 2022) [27], whereby regions with significant contributions to the energy of the ligand interaction with hot spots in the macromolecule were identified. Small organic compounds are used to map the target protein by acting as probes on its surface and locating critical regions of interest.

Hot spots have already been studied as one of the ways to identify active sites, especially in structures that do not have co-crystalized ligands. These regions act as indicators of where a drug is likely to bind. The protein structure’s cavities were further examined using Molegro software [28,29,30], bearing in mind that studies suggest that the cavities are situated near active sites.

### 3.2. Selection of Antiparasitics and Similarity of Tanimoto

Nine antiparasitic drugs were selected from the DrugBank server (https://go.drugbank.com/, accessed on 1 March 2022) [31], evaluated for their structural similarity with hydroxychloroquine, and their inhibition of SARS-CoV-2 spike protein was determined. The BindingDB server platform (http://bindingdb.org/bind/index.jsp, accessed on 5 March 2022) [32] was used to calculate the Tanimoto index as per the equation vide infra. The software utilizes merged similarity scoring, where the numbers of bits in x and y are set to 1.

$$M\left(x\right)=max_{i\in A}S\left(x,x_{i}\right)$$

The outcome rates a molecule’s similarity to another that exhibits similar properties. Therefore, the likelihood that this molecule will be active increases as *M*(*x*) increases.

### 3.3. Determination of Characteristics and Pharmacophorics

The molecules were aligned based on their Tanimoto index values using the Discovery Studio program and sent to the PharmaGist Web Server 15 (http://bioinfo3d.cs.tau.ac.il/pharma/index.html, accessed on 7 April 2022) [33,34] to determine their characteristics: Atoms (ATM), characteristics (F), spatial characteristics (SF), aromatics (ARO), hydrophobic (HYD), donors (DONN), acceptors (ACC), negatives (NEG), and positives (POS). The set used had 11 molecules based on the reference molecule (hydroxychloroquine).

### 3.4. Hierarchical Cluster Analysis (HCA) and Molecular Overlay

The pharmacophoric descriptors were employed in the adopted methodology [35] to assess their link to the Tanimoto index values, observing the significance of each factor for similarity through the Euclidean distance correlation with Minitab 19 software. The Euclidean distance, one of the most used models, is used to carry out clustering through models to identify commonalities between two points. Since *d_ab_* is the coordinate of the point in the r dimension, the Euclidean distance between points *a* and *b* can be calculated by the formula:

dab=[∑j=1p(Xaj−Xbj)2]12

where *p* denotes how many dimensions there are in space. The distance between points *a* and *b* in the coordinate system can therefore be calculated by applying the equation mentioned above. Here, the hierarchical cluster analysis (HCA) was used to verify the degree of similarity between the molecules studied.

### 3.5. Virtual Screening and Selection of Inhibitory Compounds

The 2000 molecules closest to the adopted models were chosen using the pharmacophoric characteristics generated, which included molecular weight (MW), rotatable bonds (RB), water solubility (LogS), polar surface area (PSA), number of aromatic rings (AR), hydrogen bond acceptors (HBA), and hydrogen bond donors (HBD). This virtual screening was carried out in the MolPort database using the Pharmit server (http://pharmit.csb.pitt.edu/, accessed on 7 May 2022) [36].

### 3.6. Prediction of Toxicological and Pharmacokinetic Properties

The structures obtained from the virtual screening were subjected to in silico predictions of pharmacokinetic and toxicological features, or absorption, distribution, metabolism, excretion, and toxicity (ADMET). The computations utilize physicochemical factors, drug similarity, and pharmacokinetic profiles to calculate prediction values [37,38,39]. The drugs hydroxychloroquine and chloroquine were used as a benchmark.

The following pharmacokinetic and toxicological characteristics were evaluated: Hepatotoxicity, plasma protein binding (PPB), binding to CYP2D6, aqueous solubility, polar surface area (PSA), blood–brain barrier (BBB) penetration, human intestinal absorption (HIA), Ames mutagenicity, skin irritation, eye irritation, and aerobic biodegradability.

### 3.7. Molecular Docking

This study utilized the protein structure of the SARS-CoV-2 spike receptor binding domain co-crystalized with ACE2 (Spike-ACE2) (PDB ID: 6M0J) [40] obtained from the Protein Data Bank database (https://www.rcsb.org/, accessed on 25 May 2022). The UCSF Chimera software [41] was used to remove water molecules and other residues resulting from crystallography that could interfere with the ligand–macromolecule interaction. The molecules with the best pharmacokinetic and toxicological parameters were selected for molecular docking, and hydroxychloroquine and chloroquine were used as controls.

The protein was prepared in APBS-PDB2PQR (https://server.poissonboltzmann.org/, accessed on 25 May 2022) [42], maintaining neutral pH and hence simulating the pH of the organism. The PARSE force field was used to correct the amino acid chains and adjust the conformation. Molecular docking simulations were performed through the DockThor server (https://www.dockthor.lncc.br/v2/, accessed on 26 May 2022) [43]. The grid was selected based on the x, y, and z coordinates of the active site obtained by determining the hot spots. A 20 × 20 × 20 cm cubic box was used, and the other standard parameters (number of evaluations 500,000, population size 750, and 12 runs) were used to analyze the conformations, interaction of molecules with protein amino acids, and binding energy.

### 3.8. Synthetic Accessibility Prediction and Similarity Analysis

Synthetic accessibility (SA) prediction was performed for the molecules predicted to have the best pharmacokinetic and toxicological properties through the AMBIT-AS software (http://ambit.sourceforge.net/reactor.html, accessed on 7 June 2022). This server analyzes the structural and topological characteristics of the molecules studied based on stereochemistry. The algorithm works with a score ranging from 0 to 100, where 100 symbolizes a molecule that is easily synthesized.

The similarity analysis of the molecules was evaluated with the Chemmine Tools server (https://chemminetools.ucr.edu/, accessed on 7 June 2022) through hierarchical clustering [44]. The Tanimoto index was used to calculate the similarity through the atomic descriptors through a matrix of the unique characteristics of each structure [45,46].

### 3.9. Lipophilicity and Water Solubility

The lipophilicity and solubility values were determined using the SwissADME server (http://www.swissadme.ch/, accessed on 8 June 2022) analyzing the iLOGP, XLOGP, WLOGP, MLOGP, and SILICOS-IT methods for lipophilicity and ESOL, ALI, and SILICOS-IT for water solubility, according to the methodology proposed by Sepay et al. (2020) [47].

### 3.10. Molecular Dynamics

Molecular dynamic (MD) simulation was performed for 100ns using NAMD software [48,49] in order to study the stability and binding energy of the spike protein model complexed with ligand_4, ligand_33, ligand_03, ligand_80, ligand_013. The input files were prepared using antechamber and t-leap modules of Amber 14 tools [50] and then, a 50ns MD simulation was run for five complexes along with the control compound. The LEAP program of Amber 14 [51] was employed to produce the force field, coordinate, and topology information of the complexes. Generalized Amber Force Field (GAFF) [52] was used to generate the ligand parameters, while ff14SB force field [53] was employed for protein parameters. The system was solvated by the TIP3P water model and neutralized by adding Na+ ions. The final system comprises water, ligand, and protein complexes.

To maintain the protein’s constraint at its mean position, the systems were minimized using the steepest descent minimization methodology. After that, the system’s temperature gradually increased by 300K under ensemble conditions (NPT). In the equilibrium phase, electrostatic interactions were enumerated using the particle-mesh Ewald algorithm [54] with 12 Å cutoff distance and periodic boundary conditions employed to calculate the forces of atoms, whereas all bonded interactions, such as hydrogen bonds, were constrained by the SHAKE algorithm [55]. Langevin [56] coupling was employed at constant temperature. The trajectories data were computed, and a snapshot taken at each 20ps time step.

MD trajectory was analyzed using molecular dynamic software (VMD), R-program, and Pymol. The stability and binding energy of the complexes were calculated by RMSD, RMSF, and radius of gyration. RMSD data provide average motion between coordinates.

#### Binding Free Energy Calculation

The free energy calculations were performed using the molecular mechanics energies combined with Poisson–Boltzmann (MM-PBSA) [57,58,59]. The free energy was calculated as follows:

ΔG_bind_ = ΔH − TΔS ≈ ΔE_MM_ + ΔG_solv_ − TΔS (1)

where ΔG_bind_ is the free energy of the complex, resulting from the sum of the molecular mechanics’ energy (ΔE_MM_), desolvation free energy (ΔG_solv_), and entropy (−TΔS).

ΔE_MM_ = ΔE_internal_ + ΔE_electrostatic_ + ΔE_vdW_ (2)

The energy of molecular gas phase mechanics (ΔE_MM_) can be described by the sum of the internal energy contributions (ΔE_internal_); the sum of the connection, angle, and dihedral energies; electrostatic contributions (ΔE_electrostatic_); and van der Waals terms (ΔE_vdW_).

ΔG_solv_ = ΔG_GB_ + ΔG_nonpol_ (3)

The desolvation free energy (ΔG_solv_) is the sum of the polar (ΔG_GB_) and non-polar (ΔG_nonpol_) contributions. The polar desolvation term was calculated using the implicit generalized Born (GB) approach.

g_mmpbsa was employed to investigate the binding free energy of the selected complex at the binding site of the spike protein. The binding free energy was decomposed into relative free energy of solvated complex (protein and ligand) and discrete receptor and ligand components given by the equation:

∆G_MMPBSA_ = (G_complex_ − G_protein_ − G_ligand_)

For each free energy, it is a summarization of different molecular mechanics energy including polar and non-polar solvation energy, electrostatic, and van der Waal’s contributions.

### 3.11. Determination of Theoretical Mean Inhibitory Concentration (IC_50_)

After the binding affinity values were determined, they were utilized to calculate the ligand efficiency (LE) value [23,24], where hydroxychloroquine was employed as the study’s point of reference, acting as a filter in the assessment of energies. This procedure was applied to the selected molecules; the efficiency was evaluated using the equation:

LE=−ΔG/N

where ∆G is the energy obtained from molecular docking and N is the number of non-hydrogen atoms.

To determine the average inhibitory concentration (IC_50_) for each molecule, the ligand efficiency equation vide infra was used:

$$\mathrm{LE}=1.4\times {\;\mathrm{pIC}}_{50}/N$$

where N is the number of non-hydrogen atoms, and pIC_50_ is the negative logarithm of IC_50_. Knowing the theoretical LE value through previous calculations and the molecular docking ∆G values, the formula was adjusted to obtain the pIC_50_ value and later the IC_50_ value by the following equation:

(4) $${\mathrm{pIC}}_{50}=\;\mathrm{LE}\;\times \;N/1.4$$

### 3.12. Quantum Chemical Calculations

Quantum chemical calculations were performed using the Gaussian 09 software [60]. The method adopted was the density functional theory (DFT), a functional hybrid B3LYP with the base set 6-311++g(d,p) [61,62,63]. The highest occupied molecular orbital (HOMO), lowest unoccupied molecular orbital (LUMO), and chemical descriptors of gap energy (∆E), ionization potential (PI), electron affinity (EA), and electronegativity (χ) were obtained: Chemical potential (μ), chemical hardness (η), softness (σ), and electrophilicity (ω) based on the equations below:

$$\Delta E={\;E}_{\mathrm{LUMO}}-E_{\mathrm{HOMO}}$$

$$\mathrm{PI}=-E_{\mathrm{HOMO}}$$

$$\mathrm{EA}=-E_{\mathrm{LUMO}}$$

$$\chi =-\frac{1}{2}\times \left(E_{\mathrm{HOMO}}+E_{\mathrm{LUMO}}\right)$$

μ=−χ

$$\eta =-\frac{1}{2}\times \left(E_{\mathrm{HOMO}}-E_{\mathrm{LUMO}}\right)$$

$$\sigma =\frac{1}{\eta }$$

$$\omega =\frac{\chi^{2}}{2\eta }$$

## 4. Conclusions

With this study, the probable region of the active theoretical site of the crystal structure of Spike-ACE2 was determined. Using hydroxychloroquine as a standard, we obtained the pharmacophoric model from antiparasitics. Tanimoto analysis and hierarchical analysis studies raised the hypothesis that structures with the same chemical characteristics would be great candidates for SARS-CoV-2 inhibitors.

The pharmacophores generated a list of chemical structures obtained by virtual screening that were evaluated. The molecules approved in the ADME/Tox tests showed excellent synthetic viability values, concluding that they are easy to obtain through synthesis. The pharmacokinetic and pharmacological properties showed better parameters than the control drug, and none showed carcinogenic potential, performing even better than hydroxychloroquine.

All studied molecules showed better results than hydroxychloroquine in molecular docking tests, with ligand_003 having a strong binding affinity of −8.645 kcal·mol^−1^. The lipophilicity and solubility data showed favorable values for oral administration. The theoretical IC_50_ values showed that these molecules are promising for in vitro studies and the chemical descriptors demonstrate great stability for the molecules.

## Figures and Tables

**Table 1:** Pharmacophoric model features and their coordinates.

Type		Coordinates			Radius
		X	Y	Z	
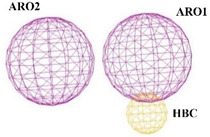	HBC	4.42	−6.72	0.20	0.50
	ARO1	4.55	−5.36	0.13	1.10
	ARO2	2.17	−5.27	−0.10	1.10

**Table 2:** Pharmacophoric characteristics of the set of molecules.

Drugs	ATM	F	SF	ARO	HYD	DONN	ACC	TI
Hydroxychloroquine	49	10	9	2	3	2	3	1.000000
Chloroquine	48	9	9	2	4	1	2	0.960265
Quinacrine	58	12	12	3	5	1	3	0.776596
Piperaquine	69	8	8	4	0	0	4	0.739884
Amodiaquine	47	10	9	3	2	2	3	0.513393
Primaquine	40	10	9	2	3	2	3	0.475962
Pyronaridine	69	15	14	4	3	2	6	0.417476
Mefloquine	42	9	7	2	2	2	3	0.359060
Quinidine	48	13	12	2	6	1	4	0.354386
Quinine	48	13	12	2	6	1	4	0.354386
Tafenoquine	61	16	15	3	6	2	5	0.328267

**Table 3:** Pearson’s correlation of characteristics selected.

Characteristics	F	SF	HYD	DONN	ACC
Spatial Features	0.973	-	-	-	-
Hydrophobic	0.709	0.713	-	-	-
Donors	0.288	0.128	0.061	-	-
Acceptors	0.766	0.732	0.161	0.106	-
Tanimoto Index	−0.547	−0.406	−0.295	−0.288	−0.564

**Table 4:** Similarity analyses by molecular overlap of drugs with the hydroxychloroquine for 100 steric and 100 electrostatic.

Drugs	Hydroxychloroquine	
	100 Steric (ste)	100 Electrostatic (ele)
Chloroquine	0.959699	0.783147
Quinacrine	0.882049	0.435233
Piperaquine	0.743533	0.597135
Amodiaquine	0.80868	0.547123
Primaquine	0.761593	0.459083
Pyronaridine	0.709077	0.350376
Mefloquine	0.772335	0.464507
Quinidine	0.813782	0.227043
Quinine	0.830381	0.449834
Tafenoquine	0.760042	0.549211

**Table 5:** Filters used in virtual sorting.

Characteristics	Minimum	Maximum
MW	259.35	535.5
RB	2.00	9.0
LogS	2.20	6.0
PSA	28.20	78.6
AR	2.00	4.0
HBA	3.00	9.0
HBD	0.00	2.0

**Table 6:** Absorption, distribution, metabolism, excretion, and toxicity (ADMET) predictions for 11 selected compounds and the control (hydroxychloroquine).

Molecules	PSA *	PPB ^a^	HepTox ^b^	CYP2D6 Binding ^c^	Solubility in Water ^d^	BBB ^e^	HIA^f^
Hydroxychloroquine	48.239	False	True	True	3	1	0
Ligand_003	61.365	True	True	False	2	1	0
Ligand_005	61.625	True	True	False	2	1	0
Ligand_013	57.788	True	True	False	2	2	0
Ligand_020	52.629	False	True	False	3	3	0
Ligand_033	64.977	True	True	False	2	2	0
Ligand_035	56.047	False	True	False	3	2	0
Ligand_044	55.985	True	True	False	2	1	0
Ligand_049	56.047	True	True	False	2	2	0
Ligand_063	61.303	False	True	False	2	2	0
Ligand_080	82.678	False	True	False	2	2	0
Ligand_086	81.353	True	True	False	2	2	0

* Polar surface area. ^a^ Plasma protein binding (PPB). ^b^ Hepatotoxicity (HepTox). ^c^ Binding to CYP2D6. ^d^ Aqueous solubility (0, good; 1, moderate; 2, poor; 3, very bad). ^e^ Blood–brain barrier (BBB) penetration (0, very high blood–brain barrier penetration; 1, high; 2, medium; 3, low). ^f^ Human intestinal absorption, HIA (0, good; 1, moderate; 2, poor; 3, very poor).

**Table 7:** Computational parameters of toxicity risk for the selected molecules.

Molecule	FDA predictions				Ames Mutagenicity	Skin Irritation	Eye Irritation	Aerobic Biodegradability
	Male Mouse	Female Mouse	Female Rat	Male Rat				
Hydroxychloroquine	Not Carcinogenic	Not Carcinogenic	Not Carcinogenic	Not Carcinogenic	Mutagen	None	Severe	Non-degradable
Ligand_003	Not Carcinogenic	Not Carcinogenic	Not Carcinogenic	Not Carcinogenic	Non-mutagenic	Soft	Moderate	Non-degradable
Ligand_005	Not Carcinogenic	Not Carcinogenic	Not Carcinogenic	Not Carcinogenic	Non-mutagenic	None	Severe	Non-degradable
Ligand_013	Not Carcinogenic	Not Carcinogenic	Not Carcinogenic	Not Carcinogenic	Non-mutagenic	None	Moderate	Non-degradable
Ligand_020	Not Carcinogenic	Not Carcinogenic	Not Carcinogenic	Not Carcinogenic	Non-mutagenic	Soft	Moderate	Degradable
Ligand_033	Not Carcinogenic	Not Carcinogenic	Not Carcinogenic	Not Carcinogenic	Non-mutagenic	Soft	Soft	Non-degradable
Ligand_035	Not Carcinogenic	Not Carcinogenic	Not Carcinogenic	Not Carcinogenic	Non-mutagenic	Soft	Severe	Non-degradable
Ligand_044	Not Carcinogenic	Not Carcinogenic	Not Carcinogenic	Not Carcinogenic	Non-mutagenic	None	Moderate	Non-degradable
Ligand_049	Not Carcinogenic	Not Carcinogenic	Not Carcinogenic	Not Carcinogenic	Non-mutagenic	Soft	Soft	Non-degradable
Ligand_063	Not Carcinogenic	Not Carcinogenic	Not Carcinogenic	Not Carcinogenic	Non-mutagenic	Soft	Moderate	Non-degradable
Ligand_080	Not Carcinogenic	Not Carcinogenic	Not Carcinogenic	Not Carcinogenic	Non-mutagenic	None	Moderate	Non-degradable
Ligand_086	Not Carcinogenic	Not Carcinogenic	Not Carcinogenic	Not Carcinogenic	Non-mutagenic	None	Moderate	Non-degradable

**Table 8:** Binding affinity and interactions of promising molecules with the Spike-ACE2 enzyme.

Molecule	∆G (kcal·mol^−1^)	Amino Acids That Interact by Hydrogen Bonding	Amino Acids That Perform Hydrophobic Interactions
Hydroxychloroquine	−7.595	ARG76, ASP73	ALA368, ASN304, GLN307, GLY172, GLY336, MET365, PHE338, PRO303, THR306, TYR173, VAL171
Ligand_003	−8.645	ASP12	ARG71, ARG76, ASN15, ASP73, GLN77, GLN78, GLU74, HIS16, LEU11, LEU123, LYS8, LYS85, PRO371, VAL75
Ligand_033	−8.303	ASP15	ARG375, ARG71, ARG76, ASP12, ASP73, GLN77, GLN78, GLU19, GLU74, HIS16, LEU11, LYS8, LYS85, PHE372, PRO371, TYR173
Ligand_013	−7.862	ARG71	ALA369, ARG76, ASN15, ASP73, GLN370, GLU19, HIS16, PHE372, PRO371, TYR173
Ligand_044	−7.749	ARG71, LYS85	ALA368, ALA369, ARG375, ARG76, ASN15, ASP73, GLN370, GLN77, GLU19, GLU74, GLY84, HIS16, PHE372, PRO371, TYR173
Ligand_080	−7.690	ASP12, GLN77, GLN78	ARG71, ARG76, ASN15, ASP73, GLU74, GLY84, HIS16, LEU11, LYS8, LYS85, PRO371, THR83, VAL75
Ligand_035	−7.635	ASN15	ARG71, ASP12, GLN77, GLN78, GLU19, GLU74, HIS16, ILE86, LEU11, LYS8, LYS85, PRO371, VAL75
Ligand_049	−7.632	ALA368, ARG375	ALA369, ARG71, ASN15, ASP73, GLN370, GLU19, GLY172, GLY336, PHE338, PHE372, PRO371, TYR173
Ligand_020	−7.508	ARG71	ALA369, ARG375, ARG76, ASN15, ASP73, GLN370, GLN77, GLU19, GLU74, HIS16, LYS85, PHE372, PRO371, TYR173
Ligand_005	−7.493	ALA368, ARG375	ALA369, ARG71, ASN15, ASP73, GLN370, GLN77, GLU19, GLU74, GLY172, GLY336, HIS16, LYS85, MET365, PHE338, PRO371, THR306, TYR173
Ligand_063	−7.394	GLU19	ARG375, ARG71, ASN15, ASP12, HIS16, LEU11, LYS8, PHE372, PRO371, TYR173
Ligand_086	−7.368	GLN307, VAL171	PHE338, PRO303, THR306, TYR173

**Table 9:** Synthetic accessibility (SA) prediction for selected compounds.

Molecule	SMILEs	SA
Ligand_020	C1=CC2=NC=C(C(=O)N3CCCCCCC3)C(=O)N2C(=C1)C	77.661
Ligand_005	N1C(SCCOc2ccccc2)=Nc3c(C1=O)c(cc(n3)C)C	75.599
Ligand_086	OC(=O)c1c2C(=O)N=C(S)N(c2nc(c1Cl)C)CCCC	75.569
Ligand_035	N1C(=Nc2nccc(-c3cscc3)c2C1=O)N4CCCCC4	75.534
Ligand_049	N1C(=Nc2nccc(-c3ccccc3)c2C1=O)N4CCCCC4	75.531
Ligand_063	Oc1c2c(nccc2-c3cccs3)nc(n1)N4CCN(C)CC4	74.658
Ligand_033	N1C(=O)c2c(ccnc2N=C1N3CCCCC3)-c4cc(OC)ccc4	74.018
Ligand_003	Oc1c2C(=S)N(C=Nc2nc(n1)N3CCC(C)CC3)c4ccccc4	73.523
Ligand_013	c1c(nc2nc(nc(N3CCCC3)c2c1C)C(=O)N4CCCCC4)C	73.432
Ligand_044	N(c1ccc(F)cc1)c2c3ccc(nc3ncc2C(=O)N4CCCCCC4)C	71.787
Ligand_080	N(C(=O)CSc1c2C(=O)N(C(=O)N(c2ncc1C(C)C)C)C)c3ccc(F)cc3	70.207
Hydroxychloroquine	OCCN(CC)CCCC(Nc1ccnc2cc(Cl)ccc12)C	69.792

**Table 10:** Lipophilicity prediction (LogPo/w) using SwissADME server.

Molecule	iLOGP	XLOGP	WLOGP	MLOGP	SILICOS-IT	Average
Ligand_003	3.01	3.38	3.11	2.73	2.99	3.16
Ligand_033	2.72	2.41	2.60	2.29	3.57	2.58
Ligand_013	3.53	3.15	2.11	2.52	3.08	2.93
Ligand_044	3.08	4.71	4.88	3.34	4.23	4.22
Ligand_080	2.44	2.72	2.85	3.17	3.22	2.67
Hydroxychloroquine	3.58	3.58	3.59	2.35	3.73	3.58

**Table 11:** Water solubility prediction (LogS) using SwissADME server.

Molecule	ESOL	Ali	SILICOS-IT	Average
Ligand_003	−4.50	−5.14	−4.65	−4.76
Ligand_033	−3.72	−3.55	−6.20	−4.49
Ligand_013	−4.03	−4.13	−4.82	−4.32
Ligand_044	−5.31	−5.66	−7,46	−6.14
Ligand_080	−4.15	−4.71	−5.94	−4.93
Hydroxychloroquine	−3.91	−4.28	−6.35	−4.84

**Table 12:** Binding free energy (kcal/mol).

S. No	Compound ID	Free Energy Value	vdW	Electrostatics	EPB	ENPOLAR
1	Hydroxychloroquine	−28.8249	−37.3748	−314.6821	328.4395	−5.2075
2	Ligand_003–spike protein complex	−26.9130	−35.2501	−1.3260	13.8309	−4.1678
3	Ligand_013–spike protein complex	−24.2420	−32.5448	−0.0613	12.3647	−4.0007
4	Ligand_033–spike protein complex	−29.94.65	−36.2508	−7.1062	17.3198	−3.9093
5	Ligand_044–spike protein complex	−30.2620	−36.0100	−3.9378	13.8663	−4.1805
6	Ligand_080–spike protein complex	−26.3248	−32.5836	−2.5741	12.5057	−3.6727

**Table 13:** pIC_50_ and IC_50_ values theoretically obtained for Spike-ACE2 targets.

Molecule	pIC_50_^a^	IC_50_^b^
Ligand_003	5.625	2.371
Ligand_013	6.232	0.586
Ligand_033	6.179	0.663
Ligand_044	5.980	1.047
Ligand_080	6.339	0.459
Hydroxychloroquine	5.139	7.268

^a^ pIC_50_ = −log(IC_50_), ^b^ IC_50_ in µM.

**Table 14:** Chemical reactivity descriptors.

Properties	Ligand_003	Ligand_013	Ligand_033	Ligand_044	Ligand_080
HOMO (ev)	−6.140	−6.334	−6.071	−6.119	−6.148
LUMO (ev)	−1.993	−1.954	−1.638	−1.939	−2.699
∆E	4.147	4.380	4.434	4.179	3.449
IP	6.140	6.334	6.071	6.119	6.148
EA	1.993	1.954	1.638	1.939	2.699
χ	4.066	4.144	3.855	4.029	4.423
µ	−4.066	−4.144	−3.855	−4.029	−4.423
η	2.074	2.190	2.217	2.090	1.725
σ	0.482	0.457	0.451	0.479	0.580
ω	3.987	3.920	3.351	3.884	5.673

## Data Availability

Not applicable.
